# Participatory development of CURA, a clinical ethics support instrument for palliative care

**DOI:** 10.1186/s12910-022-00772-1

**Published:** 2022-03-23

**Authors:** Malene Vera van Schaik, H.Roeline Pasman, Guy Widdershoven, Suzanne Metselaar

**Affiliations:** 1 Department of Ethics, Law and Humanities, Amsterdam UMC, VU University, Amsterdam, The Netherlands; 2 Department of Public and Occupational Health, Expertise Center for Palliative Care, Amsterdam UMC, VU University, Amsterdam, The Netherlands

**Keywords:** Clinical ethics support, Participatory development, New instruments, Moral resilience, Moral competences

## Abstract

**Background:**

Existing clinical ethics support (CES) instruments are considered useful. However, users report obstacles in using them in daily practice. Including end users and other stakeholders in developing CES instruments might help to overcome these limitations. This study describes the development process of a new ethics support instrument called CURA, a low-threshold four-step instrument focused on nurses and nurse assistants working in palliative care.

**Method:**

We used a *participatory development* design. We worked together with stakeholders in a Community of Practice throughout the study. Potential end users (nurses and nurse assistants in palliative care) used CURA in several pilots and provided us with feedback which we used to improve CURA.

**Results:**

We distinguished three phases in the development process. *Phase one,* Identifying Needs, focused on identifying stakeholder and end user needs and preferences, learning from existing CES instruments, their development and evaluation, and identify gaps. *Phase two,* Development, focused on designing, developing, refining and tailoring the instrument on the basis of iterative co-creation. *Phase three,* Dissemination, focused on implementation and dissemination. The instrument, CURA, is a four-step low-threshold instrument that fosters ethical reflection.

**Conclusions:**

Participatory development is a valuable approach for developing clinical ethics support instruments. Collaborating with end users and other stakeholders in our development study has helped to meet the needs and preferences of end users, to come up with strategies to refine the instrument in order to enhance its feasibility, and to overcome reported limitations of existing clinical ethics instruments.

## Introduction

Over the past decade, growing attention has been paid to the necessity and potential of including stakeholders in research aimed at improving health care [1–4]. This also pertains to instrument development studies. Participatory development involves close collaboration between researchers, end users and other stakeholders throughout every step of the research design [5]. Engaging stakeholders in the development of new instruments may result in empowering end users [6, 7], and addressing their needs adequately [8]. Furthermore, it will lead to better applicability of the instrument to its specific context [8] by early identification of problems and potential solutions [9]. Last, it helps to pave the way for successful implementation [5] and effectively results in improvement of health care processes altogether [4]. This study focuses on how a participatory approach can contribute to the development of clinical ethics support (CES) instruments. We will describe and reflect on the development of a low-threshold clinical ethics support instrument called CURA for caregivers working in palliative care.

Palliative care comes with specific and substantial moral challenges [10, 11]. These challenges are known to cause relatively high levels of moral distress, burnout symptoms and high turnover rates [12, 13]. It is important that caregivers are supported in dealing well with these challenges, both in order to provide good care, and in order to develop what is known as ‘moral resilience’, i.e. “the capacity to sustain, restore or deepen [ones] integrity in response to moral (…) distress or setbacks” [14].

In order to support caregivers in dealing with moral dilemmas in daily practice, it is essential that clinical ethics support (CES) and CES instruments respond to the wishes and needs of caregivers, and are tailored to the contexts in which they work—and the limitations and conditions that come along with these contexts [15, 16]. However, existing CES instruments have limitations that create obstacles to their use in daily (palliative) care practices. For instance, caregivers report that these instruments are often time-consuming, whereas they experience a lack of time to organize a reflection session. Furthermore, using existing instruments often need the guidance of an extensively trained facilitator or ethicist, which is not always feasible, especially in urgent situations. A third limitation is the degree of complexity, which may set a high threshold for caregivers of different educational backgrounds [15, 16].

By taking a participatory approach for the development of CURA, we sought to overcome the above mentioned limitations and to develop a CES instrument specifically tailored to support nurses and nurse assistants in palliative care. We will describe the research design in the Methods section. The development process of CURA itself is described in the Results section. In the Discussion, we reflect on this process, as well as on the question to what extent participatory development can be useful for developing CES instruments in general.

This study is conducted in the context of a national programme, funded by the Dutch government, aimed at improving the quality of palliative care in The Netherlands. In addition to this article on the development of CURA, we have also published on the content of the instrument [17], and its feasibility and first perceived effects [16].

## Method

### Research design

We used a *participatory development* design for our study. Participatory development has its roots in action research, which aims at solving problems within a specific context or community [18]. The central idea of participatory design is that if you want to create usable services or instruments, you should involve relevant stakeholders, especially the end-users [19].

In our design, we distinguished three phases. *Phase one,* Identifying Needs, focused on gaining insight in the needs and preferences of nurses and nurse assistants regarding support with moral challenges and our envisioned research design, using scientific literature. *Phase two,* Development, focused on designing, developing, refining and tailoring the instrument on the basis of iterative co-creation. *Phase three* focused on dissemination activities and planning the implementation of the final instrument in different health care settings.

We gathered our empirical data in two ways: (1) through sessions with a Community of Practice, and (2) through pilots rounds of versions the concept-instrument in education and healthcare organizations. Data collection consisted by means of audio recordings, researcher notes and questionnaires.

### Community of practice

In line with a participatory design, we worked with a *community of practice* (CoP), i.e. a group of people sharing a common interest or goal, and learn together by interacting regularly [20]. Through sharing experiences, information and recommendations, a CoP can develop new forms of practice. In this respect, a CoP goes beyond the scope of communities of interest and informal arrangements due to the fact that there is a shared goal [20]—in this case, the joint development of an ethics support instrument for palliative care.

26 stakeholders were included using purposeful sampling, among which nurses and other caregivers, nursing educators and trainers, implementation experts, managers, palliative care experts, patient organization representatives, nurses-in-training, and representatives of volunteer organizations. See Table 1 for an overview of the professions of participants and when they participated in the CoP sessions. We planned four work sessions to be the anchor and reference points throughout the study. This study was conducted prior to the COVID-19 pandemic and all sessions were meetings in real life. Meetings consisted of 3 h, and took place in approximately 6-month intervals.

**Table 1:** Members of the CoP 1

Member	1st CoP-session	2nd	3rd	4th
Specialized nurse palliative care	x		x	x
Quality manager ambulant care organisation	x	x	x	
Palliative care policy officer of a large care organisation	x	x	x	x
2 coordinators of volunteers in ambulant palliative care	x	x		x
Representative patient organization (2 persons)	x	x	x	x
Advisor training center of a large care organisation	x			
Consultant palliative care	x			
Managing director Hospice	x	x		
Lecturer of nursing, applied university	x	x	x	
Student in bioethics	x	x	x	x
Teacher vocational training (nursing)		x		
Teacher vocational training (ethics)				x
Graphic designer			x	
Manager training center		x		
Trainer nurses hematology and oncology, advisor palliative care training		x	x	x
Palliative care nurse			x	
Senior advisor palliative care cancer expertise center		x	x	x
Trainer at training center in an health care institution		x		
2 teachers advanced nursing course		x		x
2 ICU nurses				x
4 medicine students				x

During these sessions, we organized dialogues in which they could exchange views among themselves and with the researchers. Feedback on every draft version of the instrument was given, and (intermediate) results of our study were shared and discussed. Furthermore, the study design itself was discussed. For instance, which strategies could be employed in order to make the process more participatory. During these sessions, meticulous notes were made by the researchers about the process, the input and feedback given by the participants, and the joint conclusions. Based on these extensive notes, a report was made and sent to all CoP members, and the participants were given the opportunity to read and comment on these member checks of each CoP session. This was also a way of involving members who could not attend the session.

Beforehand, participants would receive the planning for the session and relevant documents. In-between work sessions, newsletters were sent to keep them updated on the progress of the study (three in sum).

## Pilots

In addition to testing the concept-instrument multiple times during the CoP sessions, we organized several pilots. In these pilots, the instrument was used and evaluated by nurses and nurse assistants. All of them were working in daily practice with patients with palliative care needs, but in diverse health care settings (academic hospital, home care, nursing homes). An overview of participants of the pilots are presented in Table 2.

**Table 2:** Overview of pilots

Pilot	Setting	Participants	Version of the instrument
1	Community of practice	10 stakeholders (see Table 1)	1
2	Training institute for continued education	± 15 registered nurses in oncology	2
3	Training institute for continued education	> 150 registered nurses and licensed nurse practitioner, multiple classes	3
3	Vocational training institute	± 20 licensed nurse practitioner in training	3
4	Health care organization providing home care and nursing home care	15 certified nurse assistants, licensed nurse practitioner and registered nurses	3

The participants of the first pilot consisted of a class of oncology nurses following a continuing education program. They tested the instrument in their own work environment and gave feedback to the researchers in class.

The second pilot group consisted of nurses following a continuing education program and working in various contexts of palliative care. After being introduced to the instrument by the researchers in class, they tested it in their own work environment and wrote an evaluation and filled in questionnaires (the results of the questionnaires are published elsewhere [16]).

The third pilot took place in another educational institute for nurses. Participants followed a part-time vocational training to become a Licensed Nurse Practitioner. They also tested the instrument in their own work environment and filled out questionnaires. Furthermore, feedback on the instrument was collected during a group discussion after the nurses had used the instrument. Of this group discussion, field notes were made by the researcher.

The fourth pilot took place within a large health care organization. We formed one group with participants working as nurses or nurse assistants in home care (n = 7) and one group working in various nursing homes (n = 8). These groups convened once a month for six times in total. During the first two meetings, the instrument was introduced and the researcher facilitated the reflection process. In the following meetings, the participants themselves used the instrument to reflect on moral challenges they experienced in practice. One of the researchers (MvS) was present at all meetings and took notes.

### Ethical considerations

Participants were aware of the scientific purposes of the data collection and gave verbal and/or written informed consent for scientific purposes. Researchers emphasized to nurses-in-training that it was not obligatory to fill in the questionnaires or to give feedback in order to pass the examination. Participation of nurses-in-training in this research design was approved by the management of the educational institutions. The healthcare professionals of the fourth testing group gave written consent for the researcher being present and making notes used for scientific purposes.

## Results

**Fig. 1:**
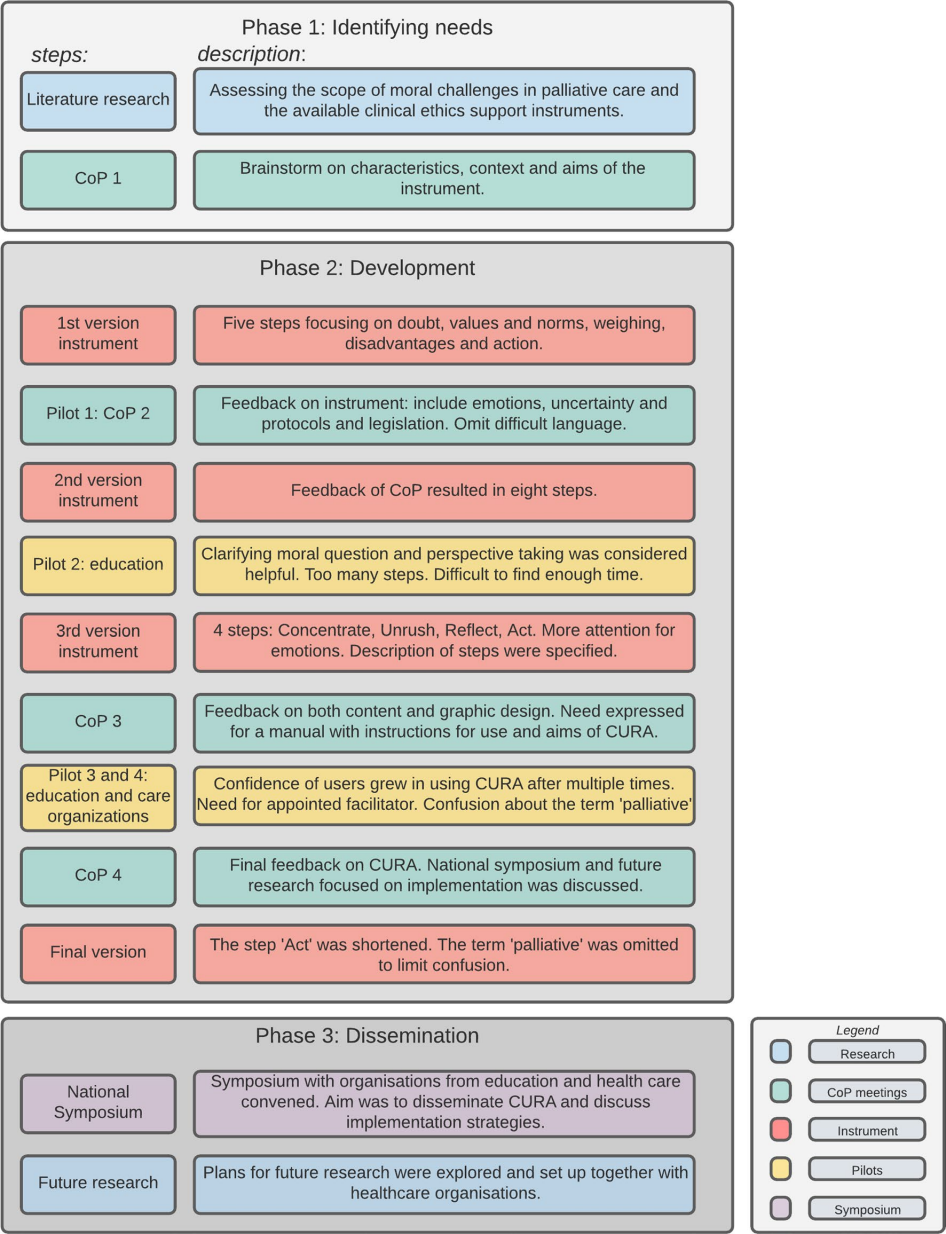
Overview of the process

In this section we will describe the process of development of CURA. The process consisted of three phases: Identifying Needs, Development and Dissemination. See Fig. 1 for an overview of the process and the steps we conducted in every phase.

### Phase 1: Identifying needs

This phase focused on the experiences and needs of (potential) end users. The researchers conducted a literature review in order to gain a general view on (1) moral challenges nurses and nurse assistants encounter in palliative care; (2) existing ethics support instruments and the perceived lacunas and/or limitations of these instruments and (3) on the envisioned research design. The insights from the literature were discussed in the first CoP.

#### 1st CoP session

All members (n = 10) of the first CoP session were informed about the aims of the research, i.e. developing a low-threshold instrument for ethics support in palliative care, and about the proposed participatory development research design.

Participants confirmed the need for low-threshold ethics support in order to deal with moral dilemmas in daily practice, and responded affirmatively to the findings of existing research on high levels of moral distress among caregivers in palliative care. Most participants indicated that ethical reflection sessions are highly useful and desirable, but take up a lot of time, oftentimes resulting in not organizing them at all. Another challenge was related to the high complexity of existing instruments, and that a trained facilitator or ethicist was needed each time to facilitate the reflection.

Subsequently, the participants were asked to reflect on what should be the characteristics and criteria of the envisioned instrument. Consensus emerged on the following aspects: (1) characteristics the envisioned instrument, (2) its context of use, and (3) its goals. The output of this first CoP is summarized in Table 3.

**Table 3:** Output 1st CoP 1

Output 1st CoP: criteria of the instrument	
Characteristics	Low in complexity Appealing and recognizable name and design No elaborate training required Central place for patient’s values Guidelines and protocols taken into account Time efficient Both individual and joint use
Context	Educational and care setting Generic: applicable in all settings of palliative care (hospice, hospital, home care and nursing homes) Usable for all educational levels of nurses and nurse assistants Applicable in daily practice No extra ‘bureaucratic burden’ Easy available
Goals	The instrument creates sensitivity and awareness of moral challenges Empowers caregivers to deal with difficult situations and moral distress Act in accordance with guidelines or deviate from them when considered justified

### Phase 2: Development

Once the needs of the stakeholders and criteria of the instrument were clear, we started the development of the instrument. The instrument was developed through cyclic-iterative co-creation, i.e. involving stakeholders in consecutive cycles of designing, piloting, and evaluating the concept-instrument.

#### Drafting the first version of the instrument

**Fig. 2:**
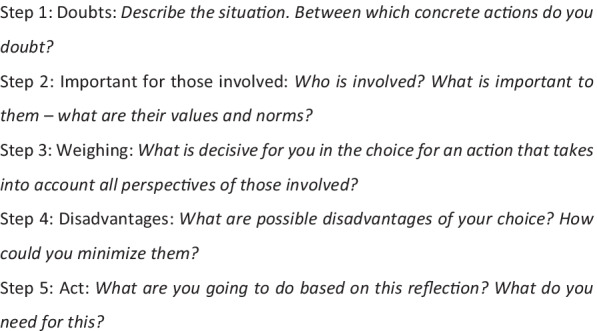
First version of instrument

We drafted a first version of the instrument prior to the second CoP session (Fig. 2). The criteria of the instrument as brought forward during the first work session of the CoP were our point of departure. In addition, we built upon prior experience with (developing) tailored ethics support instruments [15, 21] and with Moral Case Deliberation (MCD). MCD is a form of clinical ethics support, helping health care professionals to reflect systematically on their actual ethical questions and reasoning. Some key principles of MCD are: (1) taking one’s own experience as a starting point for moral reflection; (2) articulating the moral dilemma at stake; (3) exploring different moral viewpoints by venturing into the positions of stakeholders and their values and norms; (4) considering what to do in the situation at hand, and establishing a well-founded course of action. Furthermore, dialogue is highly important in MCD, as it is to foster moral learning.

These principles, together with the input of the first CoP, resulted in a draft-instrument consisting of five steps:

#### 2nd CoP session

The first draft-version of the instrument was presented and participants were asked to provide feedback. They also tested the instrument in small groups, using either a professional or personal case involving a moral challenge. The following feedback and recommendations emerged from this working session.

After testing the instrument, the participants argued that a first step should be added, in which the situation should be described. In addition, a step in which one’s first response to a situation, including emotions and physical reaction, was missed. It should also be clearer that the user is invited to reflect on what is important for themselves. Also, the patient’s perspective should be more prominent. Moreover, legislation, regulations and protocols should have an explicit place in the reflection. It was also mentioned that there should be room for uncertainty; acknowledgement for what you do not (yet) know. Finally, a moment of evaluation at the end of the steps should be included. Regarding the usage of the instrument, the CoP participants had doubt whether the instrument would be feasible for nurses with a vocational education background. Therefore, we included Licensed Nurse Practitioners in the testing grounds, to ensure their perspective was taken into account.

Furthermore, the CoP members working in education suggested testing the instrument among nurses following a continuing education program. The researchers were invited to introduce the instrument during the ethics course of the continuing education program.

#### Drafting the second version of the instrument

**Fig. 3:**
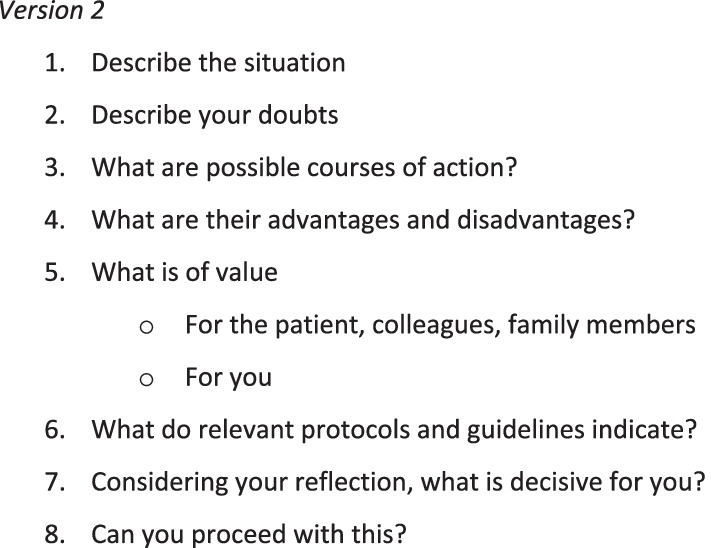
Second version of the instrument

Based on the input of the CoP we adapted the first version into the second version of the instrument (Fig. 3).

#### Piloting the second version of the instrument

Nurses-in-training received an introduction to the instrument and the study, used the instrument in class, and subsequently applied the instrument in their own work environment, involving their colleagues, and evaluated it.

What stood out in their feedback was that the instrument helps to clarify a moral question or doubt, to exchange knowledge and insights and to obtain advice from colleagues. In particular, looking at the case from the perspectives of different stakeholders (step 5) was considered supportive. Also, they found it hard to find time to use the instrument with colleagues during work hours. Step 8 ‘Can you proceed with this?’ was perceived as vague. Furthermore, eight steps was considered too much.

#### Drafting the third version of the instrument

**Fig. 4:**
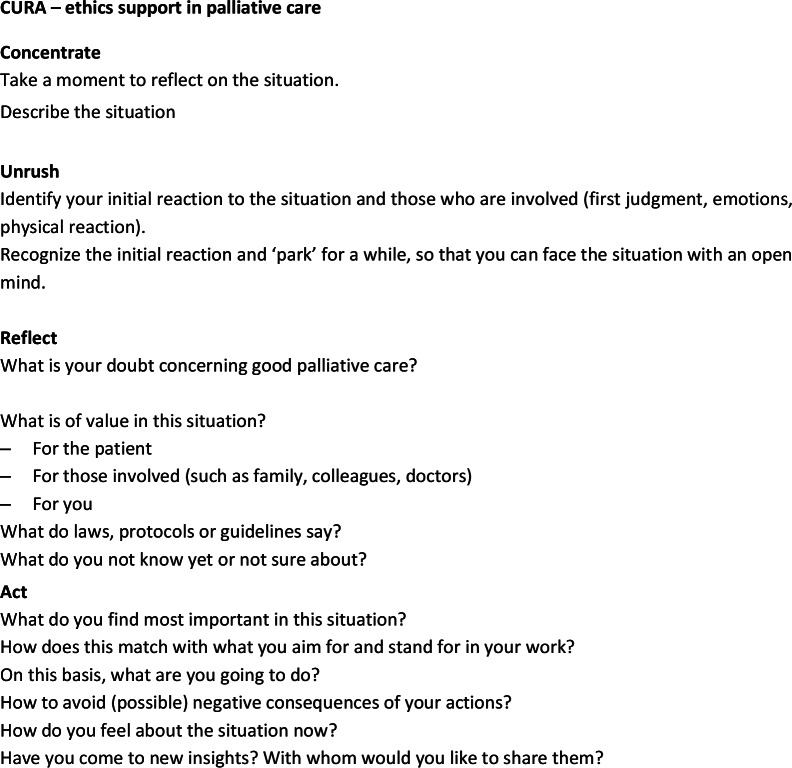
Third version of the instrument

In the third draft-version of the instrument (Fig. 4) the number of steps were reduced to four, consisting of several substeps within each step.

The first step, ‘Concentrate’, is about focusing on the situation at hand, and about zooming in on the moral doubts of caregivers. The second step, ‘Unrush’ was new. We included this extra step devoted to reflection on emotions, as it was mentioned as an important aspect by our CoP members The third step, called ‘Reflect’, ventures into what is of value of those involved in the situation. We included a substep, i.e. ‘What do you not know yet or not sure about?’. The fourth step, ‘Act’, focuses on relating moral judgment to concrete action. Here, we included a substep in which users are asked to relate their chosen course of action to their intrinsic motivation (what they aim for and stand for in their work). We changed the former step 8 into the substeps ‘How do you feel about the situation now?’ and ‘Have you come to new insights? With whom would you like to share them?’.

These four steps are an acronym of the name of the instrument, ‘CURA’. We chose the name CURA for multiple reasons, which are discussed elsewhere [17].

**Fig. 5:**
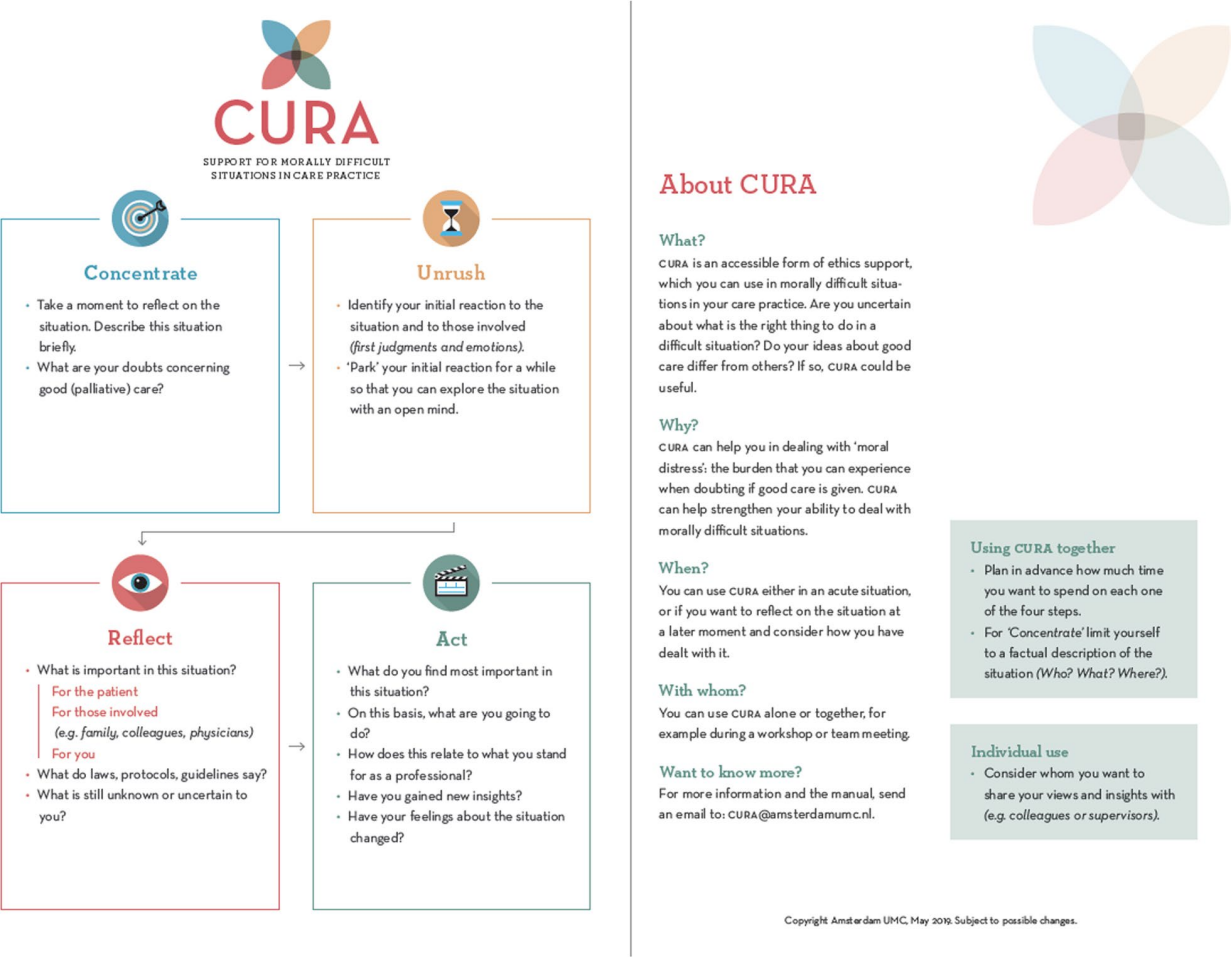
Final version of CURA

At this point, we involved a graphic designer. We informed the designer about what the CoP members considered important, such as low complexity, an appealing design, and a distinctive, recognizable logo. The lay-out should not scare off people who are not used to work with large portions of text. For the final design, see Fig. 5. We also invited the designer to the next CoP work session, so as to allow him to interact directly with the CoP members.

#### 3rd CoP session

In this session (n = 11), the third draft-instrument, was presented and tried out by the CoP members in small groups. Both content and graphic design were discussed.

Participants considered the name CURA recognizable and inviting. CoP participants concluded that CURA is relatively easy in use in a relatively short time frame (± 30 min). The step ‘Reflect’ was the most challenging, but it was argued that this is inherently so, and not due to specific formulation of the step. Participants agreed that instructions (but not too much) should be given. In addition, some short information about the goal of CURA, and when to use it, should be given in order to properly use CURA without a trained facilitator. Therefore, we drafted a first version of the manual, which provides instructions for proper usage.

#### Piloting the third version of the instrument

##### Educational setting

The third version of the instrument was used in vocational training for Licensed Nurse Practitioners and in a continuing education program. In both educational settings, the nurses-in-training combined working in practice with part-time education. Nurses-in-training received an introduction to CURA and practiced in small group setting under the supervision of the trainers. Next, the nurses-in-training were asked to use CURA in their own work environment together with their colleagues. The nurses-in-training reported about this in the following class. They were confused by the term ‘palliative’ in the instrument, wondering whether CURA was only applicable for palliative care cases. Furthermore, they mentioned that the instrument was easy in use, however, they struggled to find enough time to use the instrument together with colleagues.

##### Practical setting

In the third testing cycle we tested the instrument with nurses and nurse assistants from a large health care organization.

In particular, this group provided us with insights in what happens when CURA is used for a longer period of time (compared to the student group, who evaluated CURA after having used it only a couple of times, mostly twice). First of all, it became clear that users became more confident in using CURA, for instance in initiating and in leading the reflection as a facilitator. Furthermore, using CURA seemed to increase participant’s confidence in general. During one of the group sessions, one nurse assistant said she felt strengthened when she had to discuss a situation with the physician:

I could easily express my dilemma [to the physician], because I had already discussed the dilemma with my colleagues using CURA.

Furthermore, some participants started to use CURA when there was a moral issue within their team. It made them feel empowered and supported:

The [CURA meeting] was very good. I felt supported, like it wasn’t that weird that I had trouble accepting the situation as it was. Colleagues could see how the situation was for me, which made me feel strengthened in what I was going through.

Secondly, the researcher (MvS) remarked confusion with regard to the term ‘palliative care’, as the nurses frequently discussed whether a case that was described was indeed an ‘actual, true’ case of palliative care. In these cases, palliative care was conflated with terminal care, i.e. care at the very last stages of life, rather than care in the face of the end of life at some point.

Third, the researcher concluded that it is important to appoint a facilitator, who leads the reflection. Without an appointed facilitator, chances are that no one takes action when the reflection goes astray, or when certain steps take up too much time. Subsequently, we incorporated this in the manual.

Finally, the researcher observed an increase of reflective skills in the participants, especially among the nurse assistants, who had hardly received prior ethics education. At first, it was difficult for them to look at the case from the patient’s perspective, or to distinguish between what is important for themselves and what is important to the patient. After a couple of sessions, this started to improve.

#### 4th CoP session

In this session, results of the pilot groups were presented to the CoP members, and a final round of feedback on CURA was held. Both form and content were discussed in order to finetune and finalize the instrument (see below). The final step, ‘Act’ was considered too long and some questions were omitted, such as ‘How to avoid (possible) negative consequences of your actions?’ and ‘with whom would you like to share [your new insights]’. The tagline of the instrument was changed in order to limit confusion on the term ‘palliative care’. Participants argued for making a laminated pocket-sized card and a poster for common rooms. They also argued for a handout to be found easily online, and for inclusion of the method in existing apps and websites. Furthermore, the manual was discussed.

Also, in this session, implementation and dissemination were discussed. Participants agreed that, although CURA is to be used independently and individually without too much instruction, people could be trained to facilitate reflections with CURA, and promote their quality within their organization, so-called CURA ambassadors. Hence, they could act as ‘catalysts’ for implementation.

Third, a proposal for a national symposium on CURA was discussed. The participants agreed on making users from both care and educational settings the primary target group of the symposium and focusing on applicability of CURA in practice. The program should reflect the study’s co-creative process, as well as the fruitful interaction between teaching, research and practice.

### Phase 3: Dissemination

The final phase focused on exploring plans for dissemination and future implementation research, together with stakeholders. We concluded this development study with a national symposium. A wide variety of interested participants from health care organizations and educational settings convened. Workshops and presentations were provided by the researchers and participants from the Community of Practice, consistent with our co-creative research design. The focus of the symposium was on *disseminating* the results, *educating* people about CURA and creating a *support base* for an upcoming national implementation study.

Including an outlook on future implementation research was a pressing wish expressed by our CoP members, as they want to ensure that CURA would be sustainable: future implementation strategies was seen as an essential part of the process.

## Discussion

In this section, we will reflect on the development process, and the lessons learned about taking a participatory approach to developing CES instruments. We will conclude with an outlook on future research.

The objectives of participatory development are in line with our approach to clinical ethics support, which is rooted in philosophical pragmatism and hermeneutic ethics [21, 22]; both are aimed at a joint learning process that takes the concrete experiences and contextual knowledge of people involved as its point of departure and reference [6, 15]. What are considered to be ‘good outcomes’ cannot be determined beforehand by a priori values or aims, but should be determined *together* and should emerge from the process [5].

We consider participatory development a valuable approach for developing clinical ethics support instruments for the following reasons. First, the emergent and adaptive nature of participatory design [5, 7] and its open research design provides the possibility to utilize opportunities and carry out suggestions from stakeholders, which helps to further improve the instrument, engaging more stakeholders and expanding benefits for all involved [5, 22]. For instance, members of our CoP proposed to include a continuing education programme for nurses as a pilot setting, and to engage these nurses-in-training as co-researchers[23], thus expanding our circle of stakeholders [22].

Second, working in close collaboration with stakeholders during the development phase may help to bridge the ‘research-practice gap’ [2], i.e. the gap between researchers that produce knowledge, and practitioners who are to use this knowledge [3]. We aimed that CURA meets the needs of nurses working in palliative care and to overcome limitations that are experienced with other instruments, instrument which are perceived as time-consuming or too complex to use without guidance [15, 21]. Using a participatory development design from the onset of the development, may help to overcome these limitations. For instance, we experienced during the pilots that not all healthcare professionals are familiar with terms such as ‘dilemma’, ‘value’ and ‘norm’. Therefore, we chose not to use jargon as to ensure CURA is easy to use by all levels of healthcare professionals.

Thirdly, the involvement of stakeholders in the development of an instrument creates momentum, a sense of ownership and a network of relations that are crucial for successful implementation.

To date, little attention has been paid to describing and accounting for the way in which CES instruments are *developed*. Although some development studies have been published by our research group [15, 21], publications usually focus on describing the CES instrument *itself*, paying either limited or no attention at all to the way the instrument was created. By elaborately describing the development method and process of CURA, we seek to argue for developing CES in a participatory way in order to tailor it to the contexts, needs and wishes of its users, and to provide an example of how this can be done. Being explicit about how decisions on CES are made is important as it is the only way to be “sensitive to and consistent with the inherent characteristics of ethics (support) as a transparent, critical and deliberative professional domain”, as Schildmann et al. convincingly argue [24].

CoP participants were highly appreciative of the role of the CoP in this study. In an evaluation, they expressed that they felt being taken seriously and that they had witnessed how their input had been taken along in the subsequent steps of the process and the development of the instrument [25].

However, challenges remain. One of these challenges was stakeholder representation in the CoP. We critically reflected on this after each CoP session and made adjustments if necessary. After the first two CoPs, we noticed that we lacked ‘bedside’ nurses. For subsequent sessions we therefore invited nurses to fill this gap. This has helped to understand and respond to the needs of bedside nurses. For instance, they indicated that having the CURA method on laminated pocket-sized cards and posters for common rooms would be helpful to them, so we developed these materials.

Furthermore, we sometimes struggled with drawing joint conclusions that were truly consensus based. We had to take many perspectives, ideas and judgments into account [26]. How to avoid that some voices are heard ‘louder’ and others remain ‘dimmer’? Which power dynamics are in play in the decision-making processes [7]? In order to gain the benefits of power-sharing [5], it is imperative that researchers continuously reflect and ask feedback after each CoP session. We took this feedback into account when preparing the following CoP session. For instance, CoP-members asked for an increased focus on dialogue during the CoP sessions, as they felt the focus was too much on discussion. Therefore, we altered the set-up of the sessions by splitting up in small groups during the sessions more frequently, which fostered dialogue, rather than discussion. It also ensured that ‘dimmer’ voices were heard more equally.

In order to deal with above mentioned challenges, it is essential for researchers in participatory development to reflect on their position throughout the process [5], as the decision-making process resided ultimately with the researchers. Therefore, we kept a logbook of all feedback and input of stakeholders we received on both process and content, and we held an ongoing dialogue with our CoP participants about the choices we made [7]. This made it possible to adjust the process or the group of involved stakeholders.

A follow up study presently focuses on how CURA can best be implemented in various care settings in which palliative care is given (such as hospices, care homes, hospitals and home care). Furthermore, this follow up study assesses the effectivity of CURA once it is implemented and used on a structural basis within the organization. Does CURA indeed help to foster moral competence and moral resilience, as envisioned [17]?

CURA is specifically developed for nurses and nurse assistants working in palliative care [17]. However, other healthcare professionals and other domains of healthcare have also shown interest in CURA. More research is needed to establish whether CURA is applicable in these other contexts and if so, whether this requires adaptations to the present design.

## Conclusions

We used a participatory development design to develop a low threshold ethics support instrument for palliative care, called CURA. Working in collaboration with end users and other stakeholders has helped to meet the needs of end users, to refine the instrument and to overcome limitations of existing clinical ethics instruments. 

We have developed the instrument in three phases: (1) Identifying Needs, in which we assessed the scope of moral issues, available ethics support and needs of end users; (2) Development, in which we developed the instrument in iterative co-creation with stakeholders and (3) Dissemination, in which we paved the way for future dissemination and implementation of CURA.

Throughout the entire development process we worked with a Community of Practice, that provided us with a platform for sharing different perspectives and sources of knowledge and has created momentum and a support base for implementation of CURA.

Follow up studies focus on the implementation of CURA in different settings of (palliative) care and on the effects of CURA on the moral competences and moral resilience of health care professionals.

## Data Availability

The datasets generated and analyzed during the current study are available from the corresponding author on reasonable request. The CURA handout and manual are available free of charge from the corresponding author.
